# Effect of power ultrasound on the extraction of black caraway (*Carum carvi* L.) and evaluation of their qualitative properties using response surface methodology

**DOI:** 10.1002/fsn3.1733

**Published:** 2020-06-29

**Authors:** Dariush Shaterabadi, Mohammad Aboonajmi, Majid Ghorbani Javid, Akbar Arabhosseini

**Affiliations:** ^1^ Department of Agrotechnology College of Aburaihan University of Tehran Tehran Iran; ^2^ Department of Agronomy and Plant Breeding Sciences College of Aburaihan University of Tehran Tehran Iran

**Keywords:** ascorbic acid, DPPH, extraction, RSM, ultrasound

## Abstract

In this research, ultrasound‐assisted extraction (UAE) with two types of continuous and pulsed sonication extraction was compared with the Soxhlet method in the evaluation of qualitative traits of caraway seeds as a medicinal plant. Treatment conditions were performed at three levels of treatment (continuous and on–off levels), ultrasound power (100, 200, and 300 W), and sonication duration (10, 15, and 20min) using the response surface methodology (RSM) to compare them. Results indicated that yield of the dry extract weight and amount of ascorbic acid increased by the intensity and sonication duration directly but about DPPH radical scavenging capacity there is no direct relation, actually comparing DPPH radical scavenging capacity yields showed that it increased initially from 100W to 200 W, then from 200 to 300W, the result was reversed. It is due to the occurrence of power peaks during extraction , degradation of more and better plant matrices, and better released of the extract in low powers. Results also showed that pulsed sonication method was more effective than continuous to extraction of sensitive components.

## Graphical abstract

In this research, ultrasound‐assisted extraction (UAE) with two types of continuous and pulsed sonication extraction was compared with the Soxhlet method in the evaluation of qualitative traits of caraway seeds as a medicinal plant. According to the results of this research, higher power and sonication duration increased yield of extraction and amount of ascorbic acid.

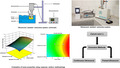

## 1 INTRODUCTION

Nowadays, extraction processes are dominant factors of production line in food, pharmaceutical, cosmetic, nutraceutic, and bioenergy industries (Chemat et al., 2017). Medicinal plants are important as one of the most important primary sources for the preparation of cosmetics, perfumery, flavoring compounds, paints, and also for the preparation of drugs. Natural products, such as spices, aromatic herbs, and medicinal plants, are complex mixtures of high‐value molecules of proven health effects (Chemat & Esveld, 2013). To exploit plant material resources, extraction techniques have been developed to obtain such valuable products commercially. These techniques are largely focused on finding technological solutions to diminish or even prevent the use of solvents in extraction processes and to obtain more highly purified products, rendering them useful in a wider range of applications (Chemat, Lagha, AitAmar, Bartels, & Chemat, 2004). Conventional solid–liquid extraction (SLE) techniques, including maceration, infusion, and “Soxhlet” extraction, are time‐consuming and use large amounts of solvents. Safety risks, toxicity of certain solvents, and presence of solvent residues in target compounds coupled with low extraction yield have stimulated interest in developing environment‐friendly (green) extraction technologies, which can minimize or eliminate the use of organic solvents. Commercial interest in more sustainable, nontoxic routes of extraction has increased, driven by growing consumer demands for greener alternatives and natural ingredients that do not involve toxic chemicals, and the environmental and health risks associated with use of chemical solvents (Tiwari, 2015).

Ultrasonic extraction is an emerging technology that can accelerate mass and heat transfer which is also an eco‐friendly method that avoids excessive use of solvents while reducing operating time. Its cavitation effect facilitates the release of extractable compounds and increases mass transfer by cell wall degradation. Several groups of food compounds such as fragrances, pigments, antioxidants, and other organic and mineral compounds can be extracted efficiently from a variety of matrices (mainly animal, food, and plant tissues) by using the ultrasonic extraction method.

Black caraway (*Carum carvi* L.) is one of the most important and valuable medicinal herbs that is wildly grown in some regions of Iran with dry weather such as Kerman, Fars, Isfahan, and Yazd provinces (Taherkhani & Nori, 2015). Black caraway is an annual herbaceous plant that belongs to Apiaceae family. Its fruits have been used as a condiment in foods and in pharmaceutical preparations to treat some gastrointestinal and inflammatory disorders. From its pharmaceutical applications, it is important to note that the antipyretic effect of this plant was reported in Avicenna's book (The Canon of Medicine) (Assami, Pingret, Chemat, Meklati, & Chemat, 2012). The caraway seeds contain essential oils rich in nutraceutical compounds used as food supplements and plant‐based medicine (Rasooli & Allameh, 2016).

In this research, extraction of caraway seeds (*C. carvi* L.) extract using ethanol–water as solvent on three levels of sonication (pulse sonication, continuous sonication, sonication duration) as following was studied. The main objective of this study was to optimize the ultrasound extraction method by introducing the pulse method and verifying it. In fact, the goal was to minimize the destruction of temperature‐sensitive compounds and reduce energy consumption.

## 2 MATERIALS AND METHODS

### 2.1 Preparation of caraway seeds

The black caraway (*C. carvi* L.) seeds were obtained from Pakan Bazr Isfahan Corporation. At first step, the seeds were ground fine to powder using grinder on 5,000 rpm (Ika Werke MF‐10 basic model). Then, 10 grams of the resulting product weighed and packaged inside aluminum foil in order to prevent the waste of active materials. In all methods, ethanol–water was used as solvent at a ratio of 6:4 (ethanol 96% and distilled water). For all treatments, the black caraway powder with a ratio of 1:10 (w/ v) to this solvent was considered. The choice of ethanol–water solvent was due to the nontoxicity of ethanol and positive effect of water on the extraction of polar compounds.

### 2.2 Ultrasound extraction

Ultrasound has been recognized for potential industrial application in the phyto‐pharmaceutical extraction industry for a wide range of herbal extracts (Vilkhu, Mawson, Simons, & Bates, 2008). For extraction by ultrasound, 100 ml laboratory glass containers were used and applied to a warm‐water bath at temperature of 40°C and extraction operation by ultrasound was performed according to the runs.

At first, the solvent was poured into the container and applied to warm‐water bath with a well‐insulated seal until it reached 40°C; then, black caraway powdered was added immediately and was stirred well.

**FIGURE 1:**
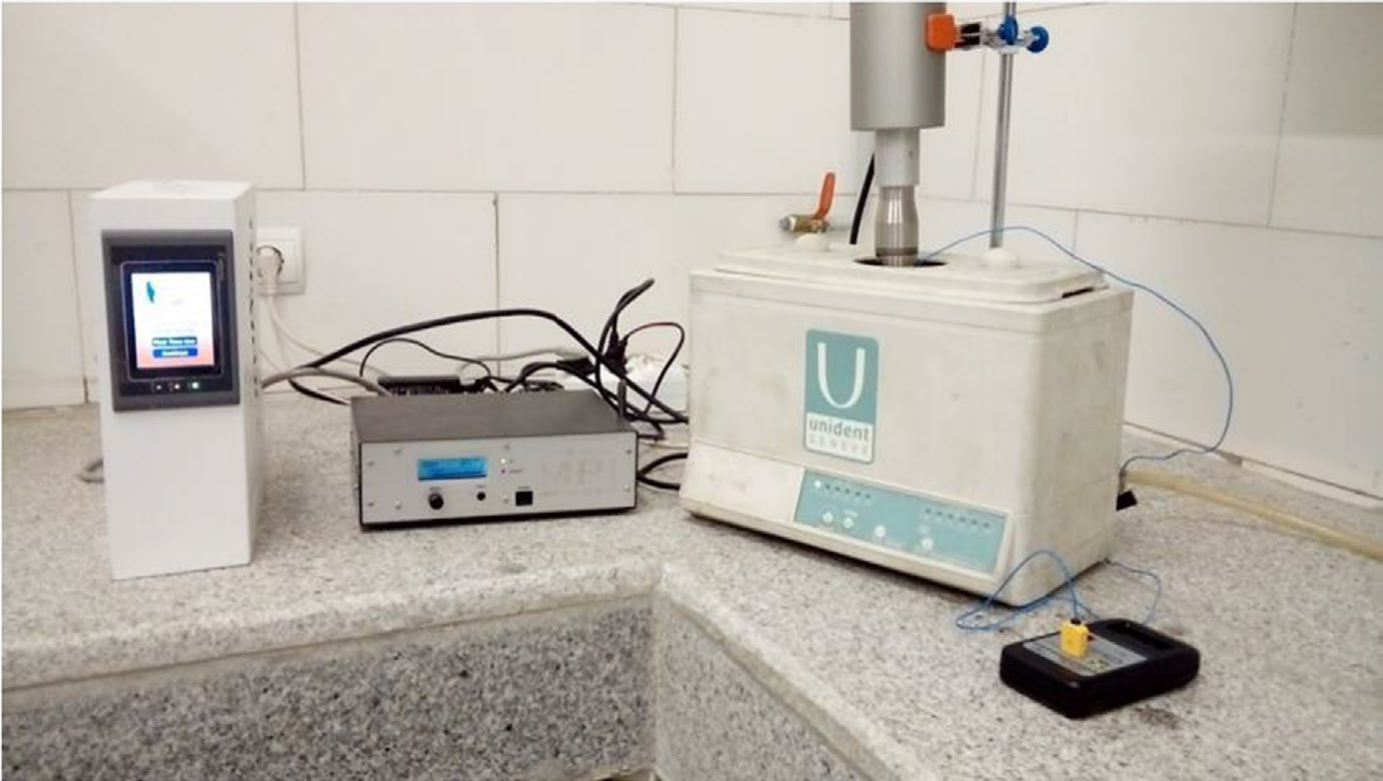
Ultrasound‐assisted extraction system

**FIGURE 2:**
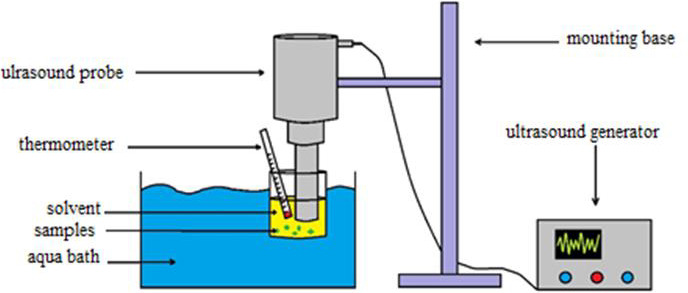
Ultrasound‐assisted extraction system schematic

Then, extraction was carried out using an ultrasound apparatus (AMMM‐1000 W, MPI‐ultrasonic) with a titanium sonotrode and 20 mm diameter. It is powered by a constant frequency of 20 ± 0.05 kHz with maximum power of 1,000 W (Figures 1 and 2).

**FIGURE 3:**
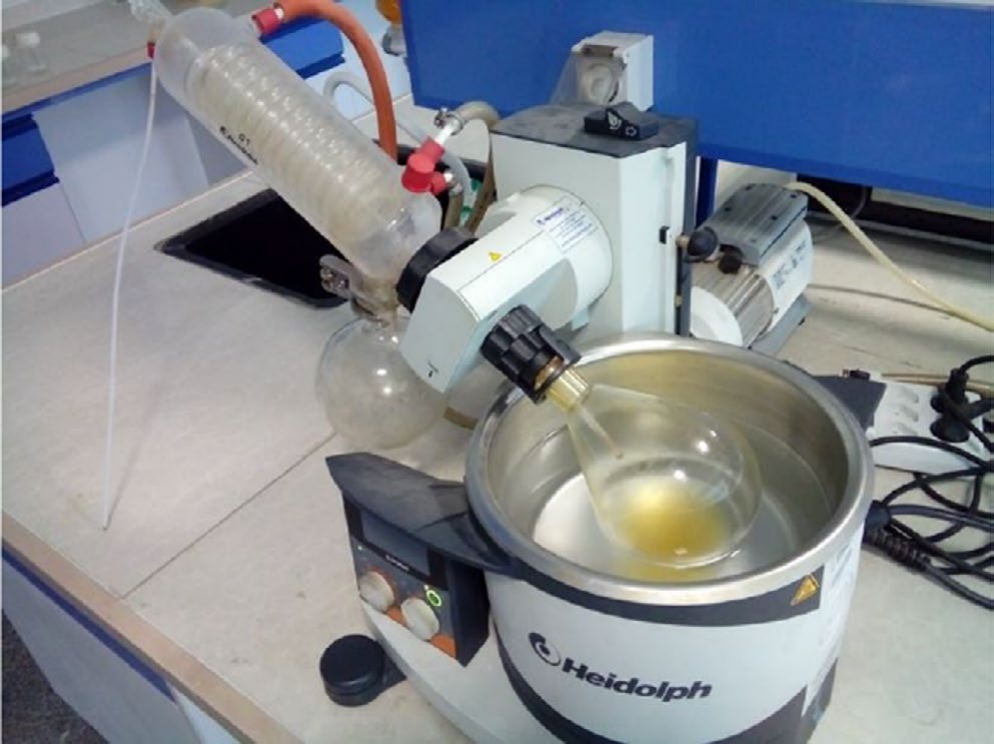
Heidolph (Beals Hei‐VAP Value) rotary evaporator

In order to separate the extracts from solids material, Whatman filter papers were used and then solvent separate from transparent extracts at 50°C and 100 ml for 15 min by Heidolph (Beals Hei‐VAP Value) rotary evaporator (Figure 3). Then, samples were dried into the oven for 3 days at 40°C completely. After drying, samples were weighed and wrapped up in aluminum foil and put in the freezer at −18°C to prevent light irritation and loss till the rest of the experiment was started.

### 2.3 Experimental design for RSM

We used RSM with central composite design (CCD) to find the optimal conditions for black caraway extraction. This research was designed and performed by Design Expert v10 software on three levels including output power intensity of ultrasound (X_1_: 100, 200, and 300W), three levels of sonication (X_2_: continuous sonication, 4s on–2s off sonication, and 2 s on–2s off sonication), and sonication duration (X_3_: 10, 15, and 20 min). The experimental ranges for the factors were determined according to the results of pretests and other researcher's results. The whole design consisted of 20 experimental points (full state), including 14 factorial points and 6 center points. Three responses including the yield of the extract (R_1_), amount of ascorbic acid (R_2_), and DPPH radical scavenging capacity percent (R_3_). For each response, the response variables were fitted to a quadratic polynomial model.

### 2.4 Ascorbic acid yield measuring method

The amount of ascorbic acid in the extract was measured by Klein and Perry's method. At first 1.5 ml of the extract was mixed with 10 ml of 1% metaphosphoric acid solution and kept in dark place for 45 min at room temperature. Then, one ml of the solution was mixed with 9 ml of solution (0.025 percent of 2,6‐dichloroindophenol, and 0.021 percent sodium bicarbonate in 100 ml of distilled water) and kept in dark place for about 15 min at room temperature.

After that, the absorbance of the solution after 15 min in 515 nm was measured by a spectrophotometer apparatus against control, which instead of one ml of extract, one ml of metaphosphoric acid solution, and 9 ml of the solution (0.225% of 2,6‐dichloroindophenol and 0.021% of sodium bicarbonate in 100 ml of distilled water) were used to prepare it (Klein & Perry, 1982).

### 2.5 DPPH radical scavenging capacity

One of the most widely used methods for evaluating the antioxidant ability is the DPPH electron transfer technique. In this method, the compound's color changed from violet to yellow by taking an electron from the antioxidant compound. The free radicals in DPPH absorb at 517 nm, which follow the Birlambert's law, and its reduction in absorption has a linear relationship with the amount of antioxidant. When a higher amount of antioxidant is added, the more DPPH is consumed and the purple color tends to be more yellowish. In this experiment, the ability to give hydrogen atoms or electron by various compounds and extracts is measured by the amount of decolorizing of violet 2,2‐diphenyl 1‐picrylhydrazyl (DPPH) in methanol (Burits & Bucar, 2000).

The antioxidant properties of the extract were measured with a little change using the Brand‐Williams et al. method: 0.1 ml of extract from each sample with 3.9 ml of methanolic DPPH solution (25 mg/L) mixed up. Then, the absorbance of the samples was read after 40 min by spectrophotometer at 515 nm wavelength. Finally, the percentage of inhibition of DPPH free radicals was calculated using the following equation (Brand‐Williams, Cuvelier, & Berset, 1995).

$$\%\text{DPPH}=\left(1-\frac{\text{samples absorbance}}{\text{control absorbance}}\right)\times 100$$

## 3 RESULT

**FIGURE 4:**
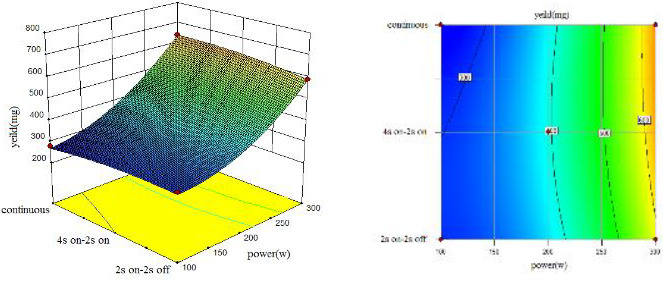
Effect of power and type of sonication on yield of extract (time = 10 min)

**FIGURE 5:**
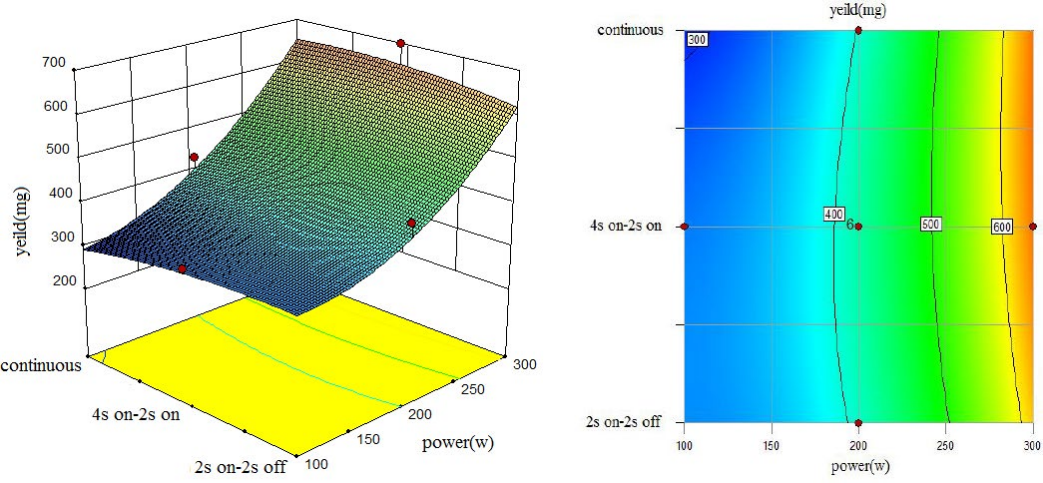
Effect of power and type of sonication on yield of extract (time = 15 min)

**FIGURE 6:**
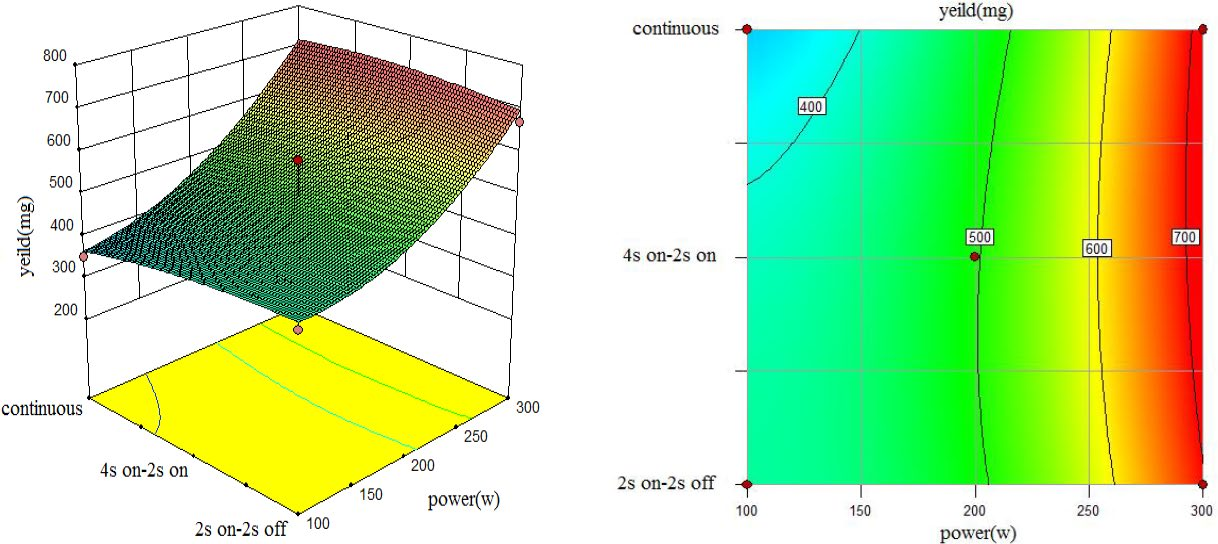
Effect of power and type of sonication on yield of extract (time = 20 min)

**FIGURE 7:**
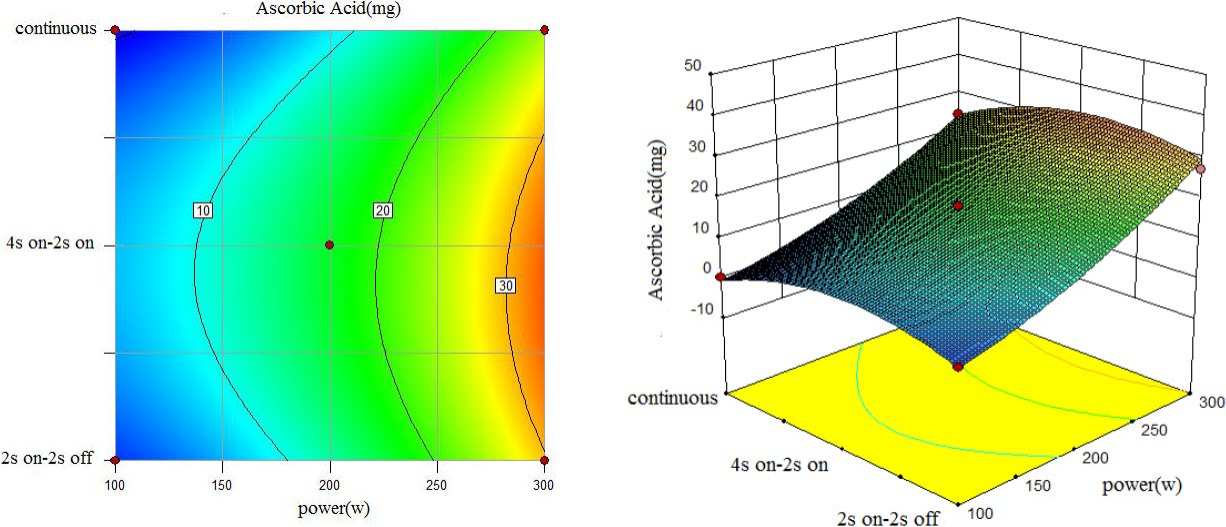
Effect of power and type of sonication on amount of ascorbic acid (time = 10 min)

**FIGURE 8:**
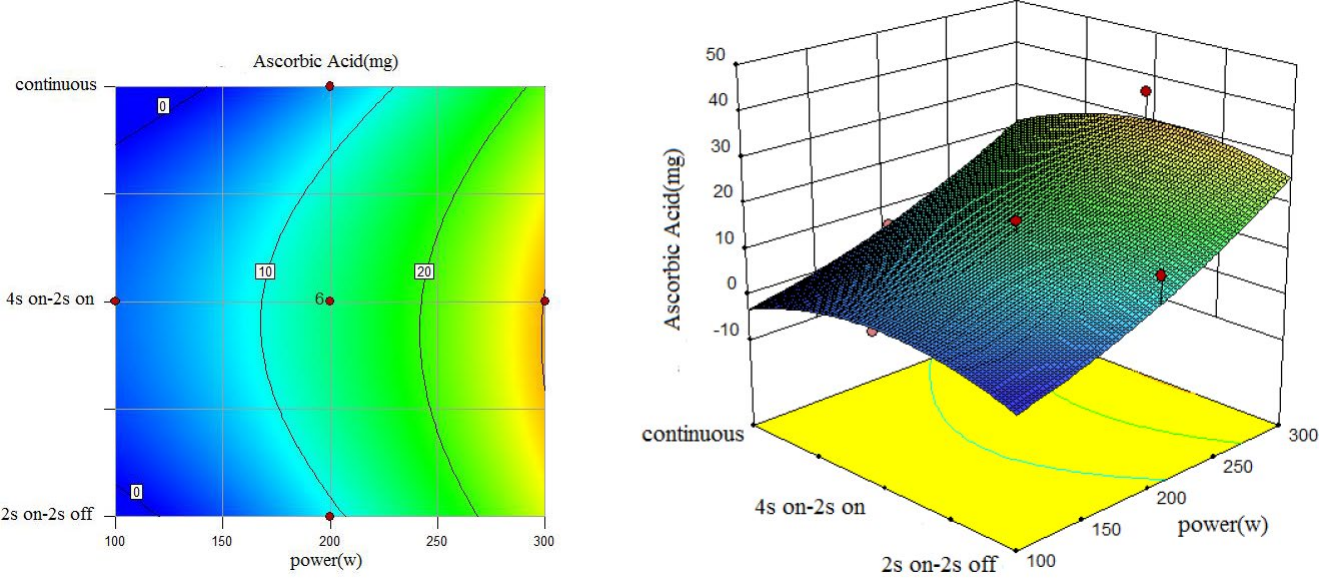
Effect of power and type of sonication on amount of ascorbic acid (time = 15 min)

**FIGURE 9:**
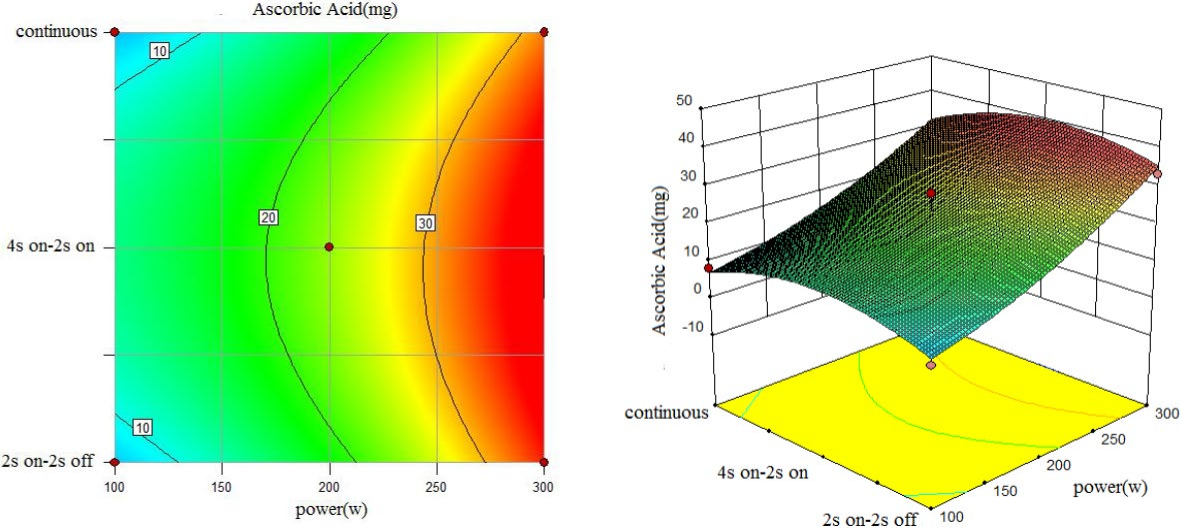
Effect of power and type of sonication on amount of ascorbic acid (time = 20 min)

**FIGURE 10:**
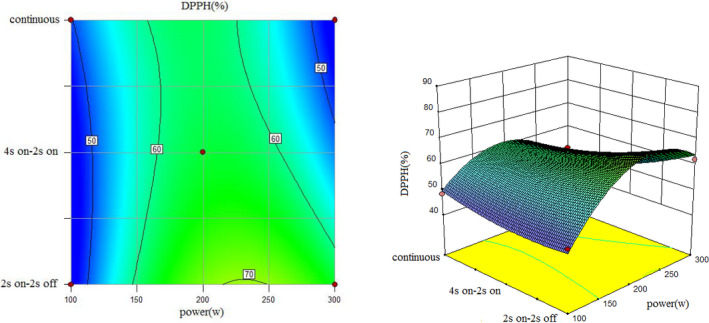
Effect of power and type of sonication on DPPH radical scavenging capacity (time = 10 min)

**FIGURE 11:**
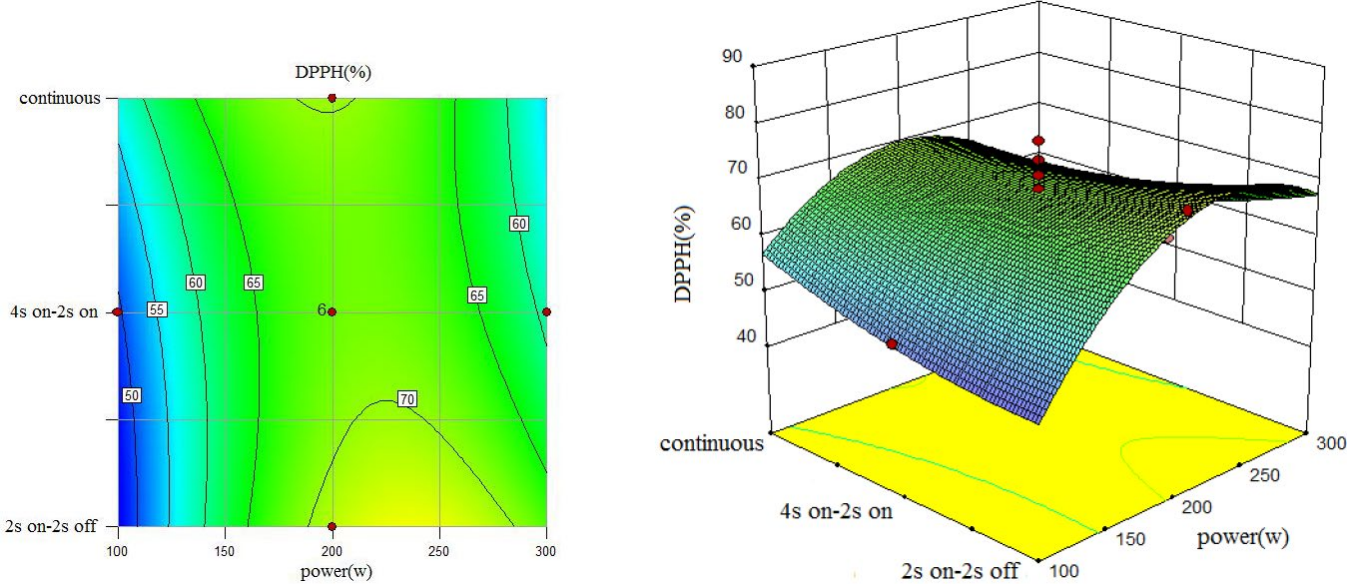
Effect of power and type of sonication on DPPH radical scavenging capacity (time = 15 min)

**FIGURE 12:**
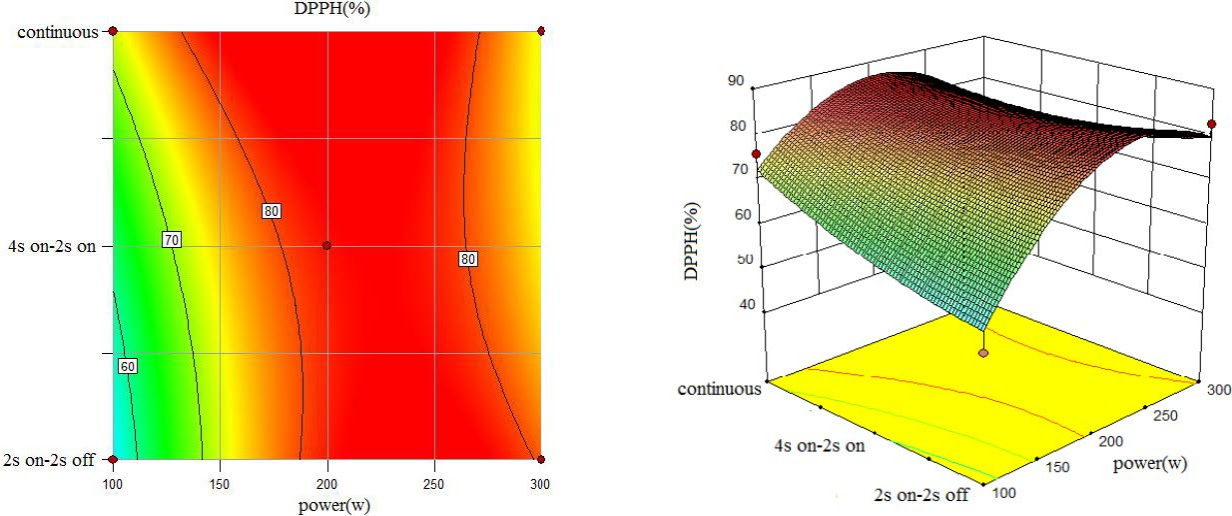
Effect of power and type of sonication on DPPH radical scavenging capacity (time = 20 min)

To understand the interaction between factors better, two kinds of response surface plot were constructed (Figures 4, 5, 6, 7, 8, 9, 10, 11, 12). The 3D graphs were generated by plotting the response using the z‐axis against two independent factors which obviously shows the effect of each factor on response. Also to find optimum values easily, 2D graphs using contour lines were plotted similarly.

### 3.1 Effect of power and sonication duration on the yield of the extract

The summary of proposed models by software for the response of yield of extract was described in the following table (Table 1), which quadratic model was chosen.

**TABLE 1:** Summary of proposed models by software for the response of yield of extract

Summary					
Source	Sequential	Lack of fit	Adjusted	Predicted	
	*p*‐value	*p*‐value	*R*‐squared	*R*‐squared	
Linear	<.0001	.0006	0.8194	0.7668	
2FI	.7850	.0004	0.7946	0.4727	
Quadratic	.0137	.0017	0.9038	0.7051	Suggested
Cubic	.3232	.0006	0.9187	−28.0121	Aliased

As shown by three following figures (Figures 4, 5, 6), the effect of power and type of excitement on the yield of the extract, the result showed that at all three levels of irradiation time, higher sonication intensity increased the yield of the extraction which were similar to the results of Caleja et al. (2017) and Da Porto, Porretto, and Decorti (2013). In general, at low power of ultrasound (100 W), pulsed sonication techniques were better than the continuous method. Results showed that 200 W sonication intensity for all treatments, yield of extraction were equal, and at 300 W, continuous sonication mode is partially had a better performance. Treatment 300 W, 15 min, and 4 s on–2 s off, and treatment 300 W, 20 min and 2 s on–2 s off, had the highest yield of extract. Comparing three forms from 10 to 20 min also showed that higher sonication duration caused a higher yield of extracts which is similar to (Bagherian, Zokaee Ashtiani, Fouladitajar, & Mohtashamy, 2011; Bonfigli, Godoy, Reinheimer, & Scenna, 2017) results.

One of the reasons for choosing the method of pulsed sonication (2 s on and 2 s off) was according to the initial experiments. It was determined that at least 2 s time needed to reach the maximum amplitude, the maximum wave intensity and wave power required, and after 2 s from the beginning of sonication, amplitude, and consequently, the intensity and power of the wave decreased and fixed on a constant value. So in this method 2 s on and 2 s off, and in general, in both on–off sonication, although the total duration of the waveform is less than that of the continuous mode, some of this times spend on the peak of amplitude, and therefore, the peak of power and intensity and the successive start and shutdown can also cause states such as shock in the solution, which could lead to more and sooner disintegration of cavitation bubbles and therefore more and better degradation of the plant matrix and hence better released of the extract in the low power which (Aguiló‐Aguayo, Walton, Viñas, & Tiwari, 2017) showed the effect of amplitude on the yield of the extract on their research.

$$\text{Intensity}=\left(\frac{\text{energy}}{\text{volume}}\right)\times \left(\text{velocity of wave}\right)=\frac{1}{2}\rho {\left(A\omega \right)}^{2}\times V_{\mathrm{wave}}$$

$$P_{m}=2\pi^{2}A^{2}f^{2}\mu V$$

which *A* is amplitude, ω is angular velocity, *P* is mean power, *ρ* is density, and *f* is frequency.

### 3.2 Effect of power and type of sonication on amount of ascorbic acid

The summary of proposed models by software for the response of ascorbic acid was described in the following table (Table 2), which quadratic model was chosen.

**TABLE 2:** Summary of proposed models by software for the response of ascorbic acid

Summary					
Source	Sequential	Lack of fit	Adjusted	Predicted	
	*p*‐value	*p*‐value	*R*‐squared	*R*‐squared	
Linear	<.0001	.0401	0.7941	0.7611	
2FI	.9727	.0227	0.7508	0.6682	
Quadratic	.0366	.0632	0.8565	0.6100	Suggested
Cubic	.5278	.0174	0.8493	−40.4956	Aliased

According to the following three figures (Figures 7, 8, 9), the effect of power and type of sonication on amount of ascorbic acid at three various time levels, it can be said that at almost all levels of time, by increasing power, amount of ascorbic acid extracted in the extract increased, and in general, 4 s on and 2 s off sonication method had better ascorbic acid yield than the continuous method and another on–off method. Comparing three forms from 10 to 20 min also showed that higher sonication duration caused a higher amount of ascorbic acid.

However, the best treatment for the highest amount of ascorbic acid in 1 cc extract was test 13 (Run8) with 200 W, 10 min and 4 s on and 2 s off, and test 10 (Run10) with 300 W, 15 min and 4 s on–2 s off had the most amount of total ascorbic acid due to the highest yield of the extract. The reason for higher ascorbic acid yield in these treatments than the continuous sonication mode can be the lower extraction temperature in these treatments than the continuous method (in principle, the disintegration of cavitation bubbles causes extraction of compounds in sonication methods, so it is possible that the very high temperature and pressure near these cavitation bubbles causes loss of acid ascorbic acid since ascorbic acid is a heat‐sensitive compound.

### 3.3 Effect of power and type of sonication on DPPH radical scavenging capacity

The summary of proposed models by software for the response of DPPH was described in the following table (Table 3), which quadratic model was chosen.

**TABLE 3:** Summary of proposed models by software for the response of DPPH

Summary					
Source	Sequential	Lack of fit	Adjusted	Predicted	
	*p*‐value	*p*‐value	*R*‐squared	*R*‐squared	
Linear	.0179	.0202	0.3564	0.0812	Suggested
2FI	.2459	.0221	0.4178	−0.2712	
Quadratic	.0125	.1026	0.7324	−0.1077	Suggested
Cubic	.3732	.0471	0.7596	−53.0034	Aliased

Regarding the following three figures (Figures 10, 11, 12), the effect of power and type of sonication on DPPH radical scavenging capacity in three levels of time can be said that although the effect of power on DPPH radical scavenging capacity percent is significant. Also, results revealed that higher sonication intensity has a nonsignificant trend on inhibitory DPPH radical scavenging capacity percent (Guss et al., 2017; Hayta & İşçimen, 2017).

However, in all methods, when power increased, at first DPPH radical scavenging capacity increased and then decreased which is similar to the result of Ghorbani, Aboonajmi, Ghorbani Javid, and Arabhosseini (2018). It means that best DPPH radical scavenging capacity performance was at 200 W  power for all methods and pulsed ultrasound (2s  on–off) and 15min sonication duration has better DPPH radical scavenging capacity compared to others. Lower DPPH radical scavenging capacity percent is due to overexposure to the radiation or thermal degradation by cavitation which similar behavior was observed in the extraction yield (Aspe, Fernandez, 2011) which (Bahmani, Aboonajmi, Arabhosseini, & Mirsaeedghazi, 2018) reported the same that higher power of ultrasonication had a destructive effect on antioxidant activity. Comparing three forms from 10 to 20 min also showed at a higher sonication duration increased DPPH radical scavenging capacity percent.

The highest DPPH radical scavenging capacity percent happened when power set on 300 W; sonication was set on 2s on–off for 20 min in pulsed mode (Run1), and the lowest DPPH radical scavenging capacity percent was observed for (Run14) when power set on 300 W; sonication was set on 4s on and 2s off periodically for 15min.

### 3.4 Optimum result for factors

According to the software optimization results, the optimum ultrasound power was 213.623 W, the optimum extraction time was 17.4018min, and the optimum sonication method was 0.403396 (which we coded three following sonication methods: continuous sonication, 4s on–2s off sonication, and 2s on–2s off sonication, respectively, 1, 0, and −1). So these means that treatment with 200 W ultrasound power, 15 min extraction time, and 4 s on–2 s off sonication had better performance among all of the treatments which were the nearest amount to the optimum results.

## 4 CONCLUSIONS

Researches proved the ultrasonic extraction method as an easy, low‐cost, fast, environmentally friendly, and high‐performance method so far, and it is an appropriate option for extraction. In order to obtain the best and most effective extraction method, it is necessary to pay attention to plant characteristics, extraction time and temperature, a suitable solvent, type of extraction method, and which compounds were more important to be obtained in the extract. Each extraction method has a different function, different compositions are obtained in the extract, and it should be paid attention that the high yield of extract does not mean a high amount of the desired compounds in the extract. Therefore, we should consider the advantages and disadvantages as well as limitations such as computational costs, waste of volatile compounds like thermos‐sensitive compounds.

According to the results of this research, higher power, and sonication duration, increased yield of extraction and amount of ascorbic acid, but in the case of DPPH radical scavenging capacity, when power increased, initially increased DPPH radical scavenging capacity and then decreased it, actually from 100 to 200 W, higher power increased DPPH radical scavenging capacity then from 200 W to 300 W, the result is reversed. This can be due to the occurrence of power peaks during extraction and, consequently, degradation of more and better plant matrices, and therefore better release of the extract in low powers. Treatment 300W power, 15min and 4s on–2 s off, and treatment 300W power, 20min and 2s on–2s off had the highest yield of the extract (700 and 670 mg, respectively). The best treatment for the highest amount of ascorbic acid in 1.5cc of the extract was treatment with 200W power, 10min and 4s on–2s off, and treatment with 300W power, 15min and 4s on–2s off also had the highest total ascorbic acid extracted, due to the highest yield of the extract. The highest DPPH radical scavenging capacity treatment was 300W power, 20 min and 2s on–2s off. According to the software optimization results, 200W ultrasound power, 15min extraction time and 4s on–2s off sonication had better performance.

## CONFLICT OF INTEREST

The authors declare that they do not have any conflict of interest.

## ETHICAL APPROVAL

This study does not involve any human or animal testing.

## INFORMED CONSENT

Written informed consent was obtained from all study participants.
